# A novel *in vitro* repellent bioassay to evaluate the effect of wood vinegar against louse *Haematopinus tuberculatus*

**DOI:** 10.1017/S0031182025000551

**Published:** 2025-08

**Authors:** Antonio Bosco, Giovanni Jesu, Alessandro Nappa, Emanuele Bambacaro, Giuseppe Martone, Serena Montagnaro, Francesco Vinale, Gabriele Panarello, Giuseppina Iacomino, Giuliano Bonanomi, Filip Strbac, Lavinia Ciuca, Laura Rinaldi, Giuseppe Cringoli

**Affiliations:** 1 Department of Veterinary Medicine and Animal Production, University of Naples Federico II, CREMOPAR, Naples, Italy; 2 Department of Agricultural Sciences, University of Naples Federico II, Naples, Italy; 3 Faculty of Technology and Innovation Science, Universitas Mercatorum, Rome, Italy; 4 Institute for Multidisciplinary Research, University of Belgrade, Belgrade, Serbia

**Keywords:** biocontrol, buffalo, *Haematopinus tuberculatus*, *in vitro* repellent bioassay, wood vinegar

## Abstract

The water buffalo farm with intensive breeding techniques promotes the spread of lice (*Haematopinus tuberculatus*), leading to a reduction in meat and milk productions. Current control measures include the use of chemicals, which in the long-term lead to the development of resistance. Given the lack of alternative solutions, the aim of this study was to evaluate the repellent effect of a low impact by-product, the wood vinegar (WV), against *H. tuberculatus* using a new *in vitro* bioassay test. The test consisted of a glass Petri dishes subdivided in 3 separate areas. WV was tested at different concentrations (100%, 75%, 50%, 25%). Each of the 3 replicates was performed with 10 adults, at 27 °C and 75% relative humidity. Observations were made 5, 15, 30, 60, 90, 120 and 180 min after application to evaluate movement, the living/dead ratio and the ability to overcome the obstacle area of the lice, in terms of comparative avoidance (%). Additional *in vitro* cytotoxicity tests were performed. The test performed showed a repellent effect of 77% for the 100% WV concentration against lice of both sexes 30 min after exposure, similar (*P* < 0.05) to the repellent effect of deltamethrin (77%). The *in vitro* bioassay developed during the experiment proved to be reliable and can be used to evaluate the repellent effect of new molecules against lice before testing them *in vivo*. Furthermore, WV has a potential to be a valid tool for the control of buffalo pediculosis, although *in vivo* studies have to be undertaken.

## Graphical abstract

**Figure:**
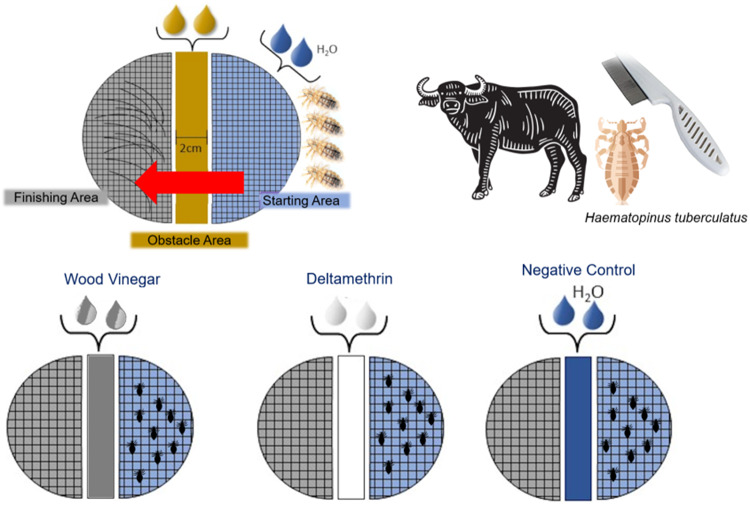

## Introduction

Sucking lice are wingless insects that live as permanent obligate ectoparasites of various mammals, including humans (Fu et al., 2022). Among these, the blood-sucking louse *Haematopinus tuberculatus* (Burmeister, 1839) is the key ectoparasite infesting the water buffalo (*Bubalus bubalis)* (L.) breeding (Shakya et al., 2022), causing direct damages characterized by various symptoms such as skin irritation, anaemia, anorexia, restlessness and loss of body condition breeding (Da Silva et al., 2013), or also indirect damages as a vector of several pathogens, including *Brucella abortus* or anaplasmosis, as observed in preliminary studies conducted by Da Silva et al. (2013) and Neglia et al. (2013), respectively. As an additive subsequent impairment attributable to lice infestations (pediculosis), there is the reduced productivity of buffalo herds, which play an important role in livestock farming in several countries (Bosco et al., 2018; Morgoglione et al., 2020). Ectoparasitic infestations occur more frequently in the winter, as animals tend to move closer together to keep warm (Shakya et al., 2022).

The Italian Mediterranean water buffalo breed is mainly reared in Italy to produce high-quality milk, mandatory for the production of the ‘buffalo mozzarella’, a fresh cheese with a protected designation of origin, according to European Union legislation (Zicarelli, 2004). Although modern intensive water buffalo farming has almost completely replaced the traditional free range/semi-wild buffalo herd and has greatly reduced helminth infections on farms, at the same time it has led to a higher incidence of protozoal and ectoparasitic diseases, due to the high density of animals (Cringoli et al., 2009; Bosco et al., 2017; Morgoglione et al., 2020).

The control of pediculosis caused by *H. tuberculatus* in buffaloes is a key issue in order to prevent economic losses (Veneziano et al., 2017). Common solutions include the use of drugs (i.e. ivermectin and eprinomectin) at the same dose and recommended intervals as those used in cattle breeding (Veneziano et al., 2013). Pyrethroid-based pesticide insecticides (deltamethrin, cypermethrin and flumethrin) are also sometimes used, but with low control effects due to the emergence of resistances in the target parasites (Shakya et al., 2022). Insects can although rapidly develop resistance to drugs due to their genetic plasticity, with rapid mutations manifested in target genes and leading to the emergence of resistant alleles, that are passed on to subsequent generations (Durand et al., 2011; McNair, 2015). The search for alternative control strategies for pediculosis in the veterinary sector is of paramount importance to limit insecticide residues in milk, environmental pollution and the emergence of resistances in target organisms, but still relatively low explored.

Recently, the wood-combustion derived products such as biochar and wood vinegar (WV) have shown promising results against many different pests in agricultural sector (Dewi et al., 2020; Bonanomi et al., 2021; Jesu et al., 2022). The WV is a by-product of wood pyrolysis and has attracted considerable interest due to its potential applications in agriculture, including plant growth promotion, and both pest and disease control (Mhamdi, 2023), which antifungal, biopreservative, antioxidant and insecticidal effects are derived from the acidic compounds, phenols and carbonyls (Faisal et al., 2019). The studies conducted have shown WV has a repellent action against numerous species of insects including flies and termites (Mhamdi, 2023; Ouattara et al., 2023), and in this study the possibility of using this product for the control of ectoparasites was explored and evaluated.

The aim of this study was to evaluate *in vitro* the possibility of using WV as a repellent against the louse *H. tuberculatus* in buffaloes, in attempt to find an alternative to chemicals. In order to evaluate the possible practical applications, WV was chemically characterized for both the organic and inorganic fractions while the cytotoxicity was assessed by cultured kidney cells. The novel *in vitro* bioassay was developed *ad hoc* during the study as a replacement for *in vivo* tests to reduce the use of animals for scientific experiments, in accordance with European regulations based on animal welfare and ethical principles (Verderio et al., 2023).

## Material and methods

### Collection of lice on the farm

Lice were collected from the skin of 22 animals using a proper comb in a commercial buffalo farm located in the Campania region, southern Italy, early in the morning and 1 h before the start of each *in vitro* bioassay replication. In this farm, no resistance of lice to pyrethroids has been recorded in the past. The lice were placed in test tubes under controlled and optimal conditions (27 °C) and transported to the entomological laboratories of the Regional Center for Monitoring Parasitic Infections (CREMOPAR, Campania Region, Italy), where the adults were selected and sexed under a stereomicroscope before the experiments.

### In vitro bioassay

**Figure 1.**
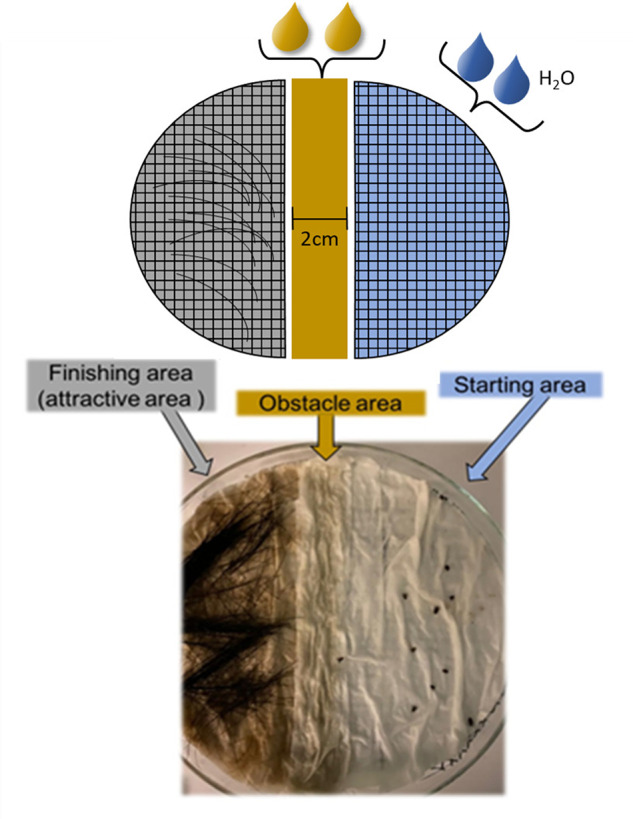
Division in 3 areas of the glass Petri dish for the *in vitro* bioassays.

**Figure 2.**
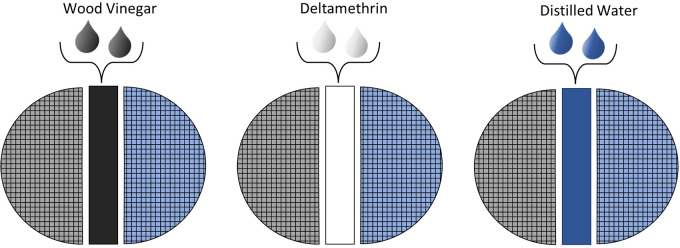
Three petri dishes to evaluate repellent effect of wood vinegar against *Haematopinus tuberculatus* confronted to negative (distilled water) and positive (deltamethrin) controls.

The *in vitro* bioassay was performed in glass Petri dishes (ø 19 cm) divided into 3 separate sections, each covered with a layer of bibulous paper soaked with 3 mL of different solutions. The first (starting) and the third (finishing) area layers were soaked with distilled water. The layer of finishing area was previously vigorously scrubbed on the host skin and then covered with buffalo fur after soaking, to increase the attraction to the lice (Figure 1). The central (obstacle) area was impregnated with different concentrations of the WV to be tested (100%, 75%, 50% and 25%). In the positive and negative controls, the central layer was soaked with deltamethrin solution (BUTOX^®^ 7,5 Pour-On, MSD Animal Health Srl) and distilled water, respectively (Figures 1 and 2).

For each biological replication, which included a test trial and 2 controls, 3 Petri dishes were set up at the same time, each with 10 adult lice (*n* = 5 males + 5 females) placed in the starting area. Experiments were carried out in a dark, controlled room at optimal condition (27 °C, 75% relative humidity), with observations made after 5, 15, 30, 60, 90, 120 and 180 min to evaluate the movement, the living/dead ratio and the overall ability to overcome the obstacle area of the lice in terms of comparative avoidance (%). In addition, *in vitro* cytotoxicity bioassay tests for all the tested concentrations were also performed, as well as a preliminary chemical characterization of WV.

### Preparation of WV and chemical characterization

The pyrolysis process took place at a facility in Ferrara (Northern Italy), using pruning residues from pome and stone fruit trees. The woody residues were cleaned from soil residues, chipped with pieces of maximum size 8 cm and subsequently subjected to pyrolysis at a temperature of 800 °C. During the process, the gases were directed into a steel pipe for condensation, and the resulting pyrolytic liquid, referred to here as WV, was collected in a plastic container for further use.

The WV was chemically characterized for the following parameters: total organic carbon (method: UNI EN 15936 2012), acetic acid (method: IS 08.03/161 2017), propionic acid (method: IS 08.03/161 2017), total phenols (method: EPA 3510C 1996 + EPA 8270E 2018), pH (method: APAT CNR IRSA 2060 Man 29 2003), electrical conductivity (method: APAT CNR IRSA 2030 Man 29 2003), total suspended solids (method: APAT CNR IRSA 2090 B Man 29 2003), ammonia nitrogen (method: UNI 11669:2017), sulphates (method: APAT CNR IRSA 4020 Man 29 2003), sulphites (method: APAT CNR IRSA 4150 Man 29 2003), sulphides (method: APHA Standard Methods 4500), chlorides (method: APAT CNR IRSA 4020 Man 29 2003), total phosphorus (method: M.U. 2252:08), nitrates (method: APAT CNR IRSA 4020 Man 29 2003), aluminium (method: EPA 3015A 2007 + EPA 6020B 2014), arsenic (method: EPA 3015A 2007 + EPA 6020B 2014), iron (method: EPA 3015A 2007 + EPA 6020B 2014), manganese (method: EPA 3015A 2007 + EPA 6020B 2014), hydrocarbons (method: UNI EN 14039:2005) and acetone (method: EPA 5021A 2014 + EPA 8260D 2018).

To extract non-polar metabolites from WV, the procedure described by Flematti et al. (2009) was followed. Briefly, 1 L of WV was extracted 3 times using 200 mL of dichloromethane (DCM) per extraction. The combined extracts were dried with anhydrous sodium sulphate (Na_2_SO_4_) and evaporated under vacuum at 40 °C using a rotary evaporator. The resulting extract was characterized via mass spectrometric analysis. For this, the DCM extract was reconstituted in methanol (MeOH) to achieve a final concentration of 1 mg mL^−1^. A 7 µL aliquot was injected into an Agilent HP 1260 Infinity Series liquid chromatograph (Agilent Technologies, Santa Clara, CA, USA) equipped with a quadrupole time-of-flight mass spectrometer (Agilent Technologies) and a diode array detector (Agilent Technologies). Sample analysis was conducted following the protocol by Staropoli et al. (2021).

The DCM extract was also analysed using gas chromatography–mass spectrometry (GC-MS). Before analysis, an aliquot of the extract was resuspended in ethyl acetate to reach a final concentration of 100 ppm and derivatized with *N,O*-bis(trimethylsilyl)trifluoroacetamide (Fluka, Buchs, Switzerland). The derivatization reaction was carried out in an ultrasonic bath (Sonorex, Bandelin Electronic GmbH & Co. K, Berlin, Germany) for 30 min. The sample was then injected into an Agilent 8890 GC system (Agilent Technologies) coupled with an Agilent 5977B MSD system (Agilent Technologies). The temperature gradient and other chromatographic and spectrometric conditions were adjusted based on the method described by Staropoli et al. (2021).

### Cell culture and cytotoxicity assay

The Madin Darby Bovine Kidney (MDBK) cells were selected as a model for the *in vitro* cytotoxicity assay. This choice is justified by the fact that this cell line originates from bovine and can therefore be considered a good model for predicting the effect of WV on buffalo host cells. In addition, MDBK cells are a well-established and widely used cell line in cytotoxicity and toxicology studies. This means that a large amount of existing data and standardized protocols are available for their use. Finally, MDBK cells are epithelial cells, which make them a suitable model for the assessment of potential toxic effects on host tissue.

MDBK cells (CCL22, American Type Culture Collection CLS Cat# 600396/p848_MDBK_(NBL-1), RRID:CVCL0421) were cultured in Dulbecco’s modified Eagle’s minimal essential medium (DMEM) supplemented with 2% fetal calf serum, 1% l-glutamine, 1% penicillin/streptomycin and 0.2% sodium pyruvate, and maintained in an incubator at 37 °C (in 5% CO_2_/95% air). Cells (2 × 10^4^ cells/well) in 96-well plates were treated at confluence with WV (0.03%, 0.1%, 0.3% or 1% in DMEM) and incubated for 24, 48 or 72 h.

Cell viability was determined using the 3-[4,5-dimethylthiazol-2-yl]-2,5-diphenyltetrazolium bromide (MTT) assay (SERVA Electrophoresis GmbH Cat# 20395.02). The principle of this method is that MTT, a soluble tetrazolium salt (3-(4,5-dimethythiazol-2-yl)-2,5-diphenyltetrazolium bromide) (5 mg mL^−1^), is converted to insoluble formazan by active mitochondrial dehydrogenases of living cells. This conversion of yellowish soluble tetrazolium into purple formazan can be determined spectrofluorimetrically (Pagnini et al., 2004). In brief, 100 μL of MTT reagent (0.5 mg mL^−1^; SERVA) was added to each well and maintained at 37 °C. After 1 h, 100 μL of solubilization buffer (dimethyl sulphoxide; SERVA Electrophoresis GmbH Cat# 39757.0) was added to dissolve the formazan crystals produced by the viable cells. After 3 h at 37 °C, the optical absorbance was measured at 570 nm using a spectrophotometer. Data are calculated as mean ± standard deviation (SEM) of 3 independent experiments performed in triplicate.

### Statistical analyses

The Cox proportional hazard model was used to evaluate the times to reach the finishing area of the treated individuals compared to the control ones (Daher et al., 2022). In the present study, this model was designed for the time to reach finishing area of the Petri dish arena, overcoming the presence of the repulsive treatment. In this context, the regression coefficient (β), standard error (SE), significance (*P*-value), relative risk (Exp(β)), as well as lower and upper limits of the confidence interval for the hazard ratio of each experimental treatment (i.e. the WV concentrations), compared to the control, are calculated. Three replicate Petri dishes, each consisting of 10 lice (experimental units), for a total of 30 experimental units, were used for each treatment.

Statistical analyses were performed with GraphPad Prism 7.0 for Windows (GraphPad Software). Statistically significant differences between the means of multiple matched groups were determined by one-way analysis of variance (ANOVA) followed by a Tukey post-hoc test. *P* < 0.05 was considered statistically significant.

## Results

### In vitro bioassay

**Figure 3.**
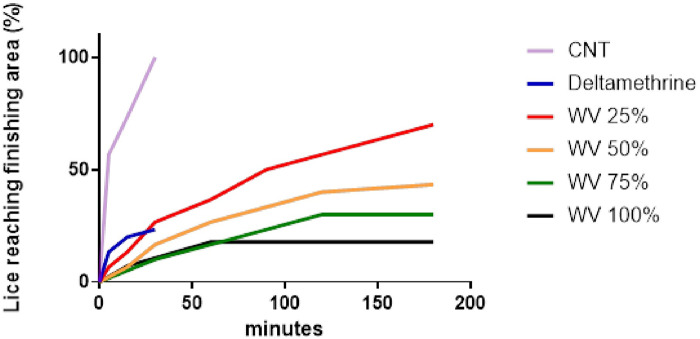
Trend of the percentage of the total lice exposed reaching the finishing area of the arena during the *in vitro* bioassay, in time. Purple (CNT): negative control, obstacle area imbue with distilled water; blue (deltamethrine): obstacle area imbue with deltamethrin solution; red (WV 25%): obstacle area imbue with wood vinegar diluted at 25% in distilled water; orange (WV 50%): obstacle area imbue with wood vinegar diluted at 50% in distilled water; green (WV 75%): obstacle area imbue with wood vinegar diluted at 75% in distilled water; black (WV 100%): obstacle area imbue with non-diluted wood vinegar. The purple line (negative control) ends before the timestone of 50.

The 100% concentration of WV had a similar repellent effect on 77% of adult *H. tuberculatus* of both sexes as deltamethrin, 30 min after exposure (*P* < 0.05). For the other WV concentrations, a dose-dependent effect was observed (Figure 3). However, deltamethrin led to the death of all exposed individuals during this period, while WV had not shown any topic effect on the tested adults, even when they stopped moving. In the negative control, all lice managed to reach the finishing area within 15 min. Regression coefficient (β), standard error (SE), significance (*P*-value), relative risk (Exp(β)) and lower and upper limits of the confidence interval for the hazard ratio of each experimental treatment, compared to the control, are presented in Table 1. The negative β values, derived from the statistical analysis, confirm the direct effect of the treatments on the increasing difficulty of overcoming the repulsive effect of WV and reaching the finishing area. In addition, the *in vitro* bioassay cytotoxicity tests showed no toxic effects.

**Table 1.** Summary of cox proportional hazard model for time to reaching the finishing area of the Petri dish arena, overcoming the presence of the repulsive treatment

					95% CI for Exp(β)	
CNT vs	β	SE	Sign. *P*-value	Relative risk of reaching the finishing area Exp(β)	Inferior	Superior
WV25%	−0.077	0.016	<0.001	0.926	0.897	0.956
WV50%	−0.049	0.010	<0.001	0.952	0.934	0.970
WV75%	−0.040	0.008	<0.001	0.961	0.945	0.976
WV100%	−0.033	0.007	<0.001	0.967	0.953	0.981

WV, wood vinegar; β, regression coefficient; SE, standard error; CI, confidence interval.

**Figure 4.**
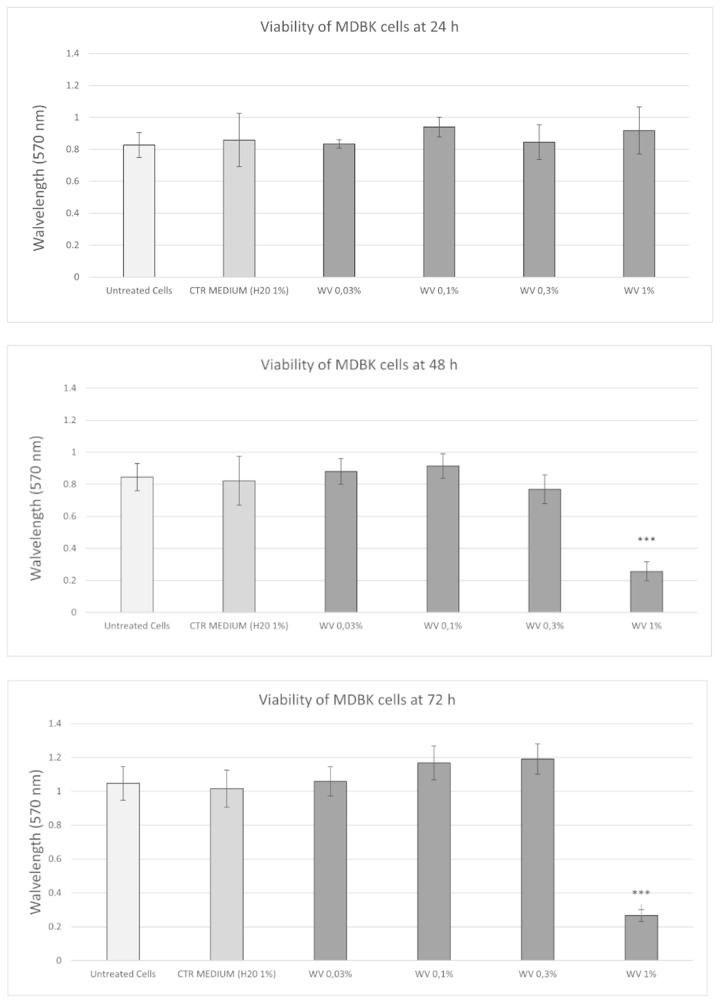
The MTT test was performed at different hours after treatment and absorbance was measured as described in the Materials and methods section. The results are presented as mean ± SD of 3 independent experiments performed in triplicate. Statistical differences between untreated cells and treated groups were analysed by One-way analysis of variance (ANOVA) followed by Tukey post-hoc test and expressed as probability *P*. *P < 0.05, **P < 0.01 and ***P < 0.001.

To determine the effect of WV on cell viability of the MDBK cell line, the MTT assay with increasing concentrations (0.03%, 0.1%, 0.3% and 1%) of WV for 24, 48 and 72 h was performed. In MDBK, cell viability decreases only at a concentration of 1% WV and after 48 and 72 h (*P* < 0.001) (Figure 4).

### WV chemical characterization

The WV was acidic (pH 3.2) and had an electrical conductivity of 1389.10 μS cm^−1^. The phenol concentration was high (54.00 mg L^−1^), as the concentration of acetic acid (27 840.16 mg L^−1^), and propionic acid (72 447.06 mg mL^−1^). The total suspended solids were 26.00 mg L^−1^, ammonium nitrogen was 8.30 mg L^−1^, while sulphates, sulphites and sulphides were 53.70, 0.32 and 0.12 mg L^−1^, respectively. Chlorides were 29.20 mg L^−1^, total phosphorus 0.97 mg L^−1^, nitrates 5.20 mg L^−1^, aluminium 0.92 mg L^−1^, arsenic 0.02 mg L^−1^, iron 183.00 mg L^−1^, manganese 1.11 mg Lv, total organic carbon 15 600.00 mg L^−1^, hydrocarbons 77.10 mg L^−1^ and acetone 320.00 mg L^−1^ (Table 2).

**Table 2.** Chemical and biochemical parameters of the wood vinegar

Parameter	Unit	Result
Acetic acid	mg mL^−1^	27 840.1
Propionic acid	mg mL^−1^	72 447.0
Phenols	mg mL^−1^	54.1
pH	U.pH	3.2
Electrical conductivity	µS cm^−1^	1389.1
Total suspended solids	mg L^−1^	26.0
Ammonia nitrogen	mg L^−1^	8.3
Sulphates	mg L^−1^	53.7
Sulphites	mg L^−1^	0.32
Sulphides	mg L^−1^	0.12
Chlorides	mg L^−1^	29.2
Total phosphorus	mg L^−1^	0.97
Nitrates	mg L^−1^	5.2
Aluminium	mg L^−1^	0.92
Arsenic	mg L^−1^	0.02
Iron	mg L^−1^	183
Manganese	mg L^−1^	1.11
Total organic carbon	mg L^−1^	15 600.4
Hydrocarbons	mg L^−1^	77.1
Acetone	mg L^−1^	320.0

Additional analysis of the organic extract using LC-MS identified 54 compounds, with 2 of them putatively matched to an internal database of plant secondary metabolites. These were identified as nicotinic acid (also referred to as niacin or vitamin B3) and malvalic acid. The DCM extract of the WV was further analysed using GC-MS, which revealed 133 chromatographic peaks. Of these, 23 compounds were identified through comparison of their mass spectra with the NIST library and verification of their retention indices (Table 3). The detected compounds spanned several categories, including phenolic derivatives (e.g. guaiacol, syringol, eugenol, catechol), fatty acids (such as oleic, stearic and palmitic acids), polyalcohols (glycerol) and pyrimidine derivatives (2,5-dimethyl-4-pyrimidinamine).

**Table 3.** Wood vinegar compounds identified by GC-MS analysis

	RT (min)	RI
Phenol, TMS derivative	5.617	1064.9
*o*-Cresol, TMS derivative	6.673	1140.4
*m*-Cresol, TMS derivative	6.8	1149.4
*p*-Cresol, TMS derivative	6.938	1162.6
Guaiacol, TMS derivative	8	1234.6
Diethylene glycol, 2TMS derivative	8.231	1250.8
Glycerol, 3TMS derivative	8.699	1283.8
2-Methyl-4-*oxo*-3,4-dihydro-2*H*-pyran-3-yl isobutyrate	8.834	1293.3
Catechol, 2TMS derivative	9.279	1326.3
2,5-Dimethyl-4-pyrimidinamine, TMS derivative	9.575	1348.7
4-Methylcatechol, 2TMS derivative	10.242	1399
Vanillin, TMS derivative	10.271	1402
Syringol, TMS derivative	10.374	1409.9
Protocatechuic aldehyde, 2TMS derivative	11.141	1472.9
4-Ethylsyingol, TMS derivative	12.035	1564.3
Eugenol, TMS derivative	12.161	1578.6
*m*-Ethoxycarbonylaniline	12.301	1594.7
2-Methoxymandelic acid, ethyl ester, TMS derivative	13.429	1776
Syringaldehyde, TMS derivative	13.785	1849.2
Vanillic Acid, 2TMS derivative	13.838	1860.8
Propyl vanillate, TMS derivative	14.586	2044.3
Palmitic acid, TMS derivative	15.24	2223.5
Oleic acid, (*Z*)-, TMS derivative	15.301	2239.2
Stearic acid, TMS derivative	16.085	2428.2

RT, retention time; RI, retension index.

## Discussion

The present study is the first to demonstrate the potential use of WV against ectoparasites in buffaloes. Specifically, the results of the *in vitro* bioassay highlight WV’s repellent effect on *H. tuberculatus* without the necessity to kill them, a species with reported resistance to the commonly used pesticide deltamethrin (Shakya et al., 2022). Considering the well-documented role of lice as vectors for certain diseases (Da Silva et al., 2013; Neglia et al., 2013) and their widespread distribution (Štrbac et al., 2023), controlling these ectoparasites in farms has proven to be more critical than previously thought. Moreover, exclusive reliance on ectoparasiticides poses significant risks to public health due to residue accumulation in animal-derived products and potential environmental contamination. In this context, natural products like WV offer a more sustainable and safer alternative (Sarfraz et al., 2023; Baz et al., 2024; Ratajac et al., 2024), as their biodegradability and low toxicity demonstrated in cytotoxicity tests make them suitable for integrated parasite management.

The WV has garnered significant attention not only for its efficiency in control pests and diseases but also for its positive effects in agriculture, such as promotion of plant growth (Mhamdi, 2023). Its ability to stimulate soil microbial activity while inhibiting pathogen growth is largely attributed to its antimicrobial compounds, particularly acetic acid. The high concentrations of acetic and propionic acid in WV have recently been exploited for the control of weeds (Liu et al., 2021). Furthermore, the presence of nicotinic acid in WV connects its applications to pharmaceutical and commercial uses aimed at improving mammalian health (Rasheed et al., 2023; Yang et al., 2024). The sulphate content in WV, known to affect the biology of species like *Daphnia magna* (Palma et al., 2009) and *Myzus persicae* (Douglas, 1988), may also influence *H. tuberculatus*, though this requires further investigation. Additionally, the high volatility and significant repellent properties of methyl isoeugenol (Scolari et al., 2021) known to deter pests across various orders (He et al., 2023; Niassy et al., 2023; Santos et al., 2023) enhance WV’s potential. Propionic acid, the most abundant compound in WV, is a well-known lethal and repellent agent effective against ectoparasites and useful in packaging to prevent infestations (Germinara et al., 2010). Its dose-dependent effects, as demonstrated in this study, align with findings from similar natural products (Germinara et al., 2007).

The WV has also shown promising results against mosquitoes, with lethal effects reported by Paulraj et al. (2011). Its dose-dependent properties are consistent with those observed in other natural extracts (Malheiro et al., 2016; Bonanomi et al., 2021) supporting its versatility. Additionally, WV has been studied for wood preservation (Akkuş et al., 2022), its efficacy against stored-product pests (Hashemi et al., 2014; Othman et al., 2023) and general in pest control (Kim et al., 2008; Kiarie-Makara et al., 2010). In a recent study, Jesu *et al*. (2023) demonstrated that a similar by-product could alter the fitness of *Bactrocera oleae* on field and also the fly microbiome upon ingestion, despite its congenital plasticity (Jesu et al., 2024), suggesting that similar effects might occur in *H. tuberculatus*. However, further studies are needed to confirm this, given the differences in feeding behaviour between the 2 species. Derivatives from biochar production have shown broad pesticidal (Bonanomi et al., 2021; Jesu *et al*., 2023; Oramahi et al., 2023) and defensive (Chenari *et al*., 2023; El-Fawy et al., 2023; Gama et al., 2023) potential, often with low environmental impact while supporting agricultural systems (Darwesh and Elshahawy, 2023; Essa et al., 2023). These characteristics make WV an excellent candidate for integrated parasite management strategies under both controlled and field conditions. A recent study by Iacomino et al. (2024) further explored WV’s multifaceted potential, investigating its bioactivity in various settings, including field trials, greenhouse experiments and *in vitro* assays: in olive groves, WV failed to exhibit repellent effects against *B. oleae* when applied as an aerosol bu, conversely, field trials showed that a 1% WV concentration significantly reduced *Meloidogyne incognita* nematode infections in strawberry plants by 15%. These findings underscore the critical need to optimize WV’s concentration and application methods to maximize its benefits while minimizing adverse effects. WV’s varying efficacy across different scenarios highlights its adaptability, serving both as a pest control agent and a soil conditioner and growth promoter. With its abundant bioactive compounds and environmentally friendly production, WV aligns with the principles of a circular economy, presenting a valuable resource for organic and integrated farming systems. The results of the present study confirm the hypothesized repellent effect of WV against *H. tuberculatus* and indicate the possibility of using this natural product to control pediculosis in buffaloes. In addition, the *in vitro* bioassay developed proved to be reliable in evaluating the repellent effect of molecules against lice. However, despite the *in vitro* bioassay cytotoxicity tests showed no toxic effects, further and thorough analyses must be carried out to better understand the mechanisms of action before an actual and safe use of WV on field.

## References

[ref1] Akkuş M, Akçay Ç and Yalçın M (2022) Antifungal and larvicidal effects of wood vinegar on wood-destroying fungi and insects. *Maderas Cienc. Tecnol* 24, doi:10.4067/s0718-221x2022000100437.

[ref2] Baz MM, Alfagham AT, Al-Shuraym LL and Moharam AF (2024) Efficacy and comparative toxicity of phytochemical compounds extracted from aromatic perennial trees and herbs against vector borne *Culex pipiens* (Diptera: *Culicidae*) and *Hyalomma dromedarii* (Acari: *Ixodidae*) as green insecticides. *Pakistan Veterinary Journal* 44, 55–62. doi:10.29261/pakvetj/2024.144.

[ref3] Bonanomi G, Zotti M, Cesarano G, Sarker TC, Saulino L, Saracino A, Idbella M, Agrelli D, D’Ascoli R and Rita A (2021) Decomposition of woody debris in Mediterranean ecosystems: The role of wood chemical and anatomical traits. *Plant & Soil* 460, 263–280. doi:10.1007/s11104-020-04799-4.

[ref4] Bosco, A, Morgoglione, ME, Amadesi, A, Masiello, I, Antenucci, P, Cringoli, G, Rinaldi, L (2018) Efficacy of deltamethrin pour-on (BUTOX® 7.5 Pour-On) against *Haematopinus tuberculatus* in Italian Mediterranean Buffalo (*Bubalus bubalis*). *Large Animal Review* 24 (2), 73–79.

[ref5] Bosco A, Rinaldi L, Maurelli MP and Cringoli G (2017) Parasitological scenario of Buffalo farms in central and southern Italy: A review. *The Buffalo (Bubalus Bubalis) – Production and Research* 298–312. doi:10.2174/9781681084176117010014.

[ref6] Cringoli G, Musella V, Maurelli MP, Morgoglione ME, Santaniello A, Condoleo R, Guariglia I and Rinaldi L (2009) Helminths and arthropoda in buffalo farms from the Lazio region (Italy). *Veterinary Research Communications* 33(Suppl 1), 129–S131. doi:10.1007/s11259-009-9268-6. 19588261

[ref7] Daher M, Varghese J, Gruschkus SK, Jimenez C, Waguespack SG, Bedrose S, Altameemi L, Bazerbashi H, Naing A, Subaiah V, Campbell MT, Shah AY, Zhang M, Sheth RA, Karam JA, Wood CG, Perrier ND, Graham PH, Lee JE and Habra MA (2022) Temporal trends in outcomes in patients with adrenocortical carcinoma: A multidisciplinary referral-center experience. *The Journal of Clinical Endocrinology & Metabolism* 107(5), 1239–1246. doi:10.1210/clinem/dgac046. 35092681 PMC9016449

[ref8] Darwesh OM and Elshahawy IE (2023) Management of sunflower charcoal-rot and maize late-wilt diseases using the aqueous extract of vermicompost (vermitea) and environmental-safe biochar derivative (wood vinegar). *Scientific Reports* 13, 17387. doi:10.1038/s41598-023-43974-2. 37833470 PMC10575965

[ref9] Da Silva AS, Lopes LS, Diaz JDS, Tonin AA, Stefani LM and Araújo DN (2013) Lice outbreak in buffaloes: Evidence of *Anaplasma marginale* transmission by sucking lice *Haematopinus tuberculatus*. *Journal of Parasitology* 99, 546–547. doi:10.1645/GE-3260.1. 23050728

[ref10] Dewi R, Hastuti N and Nuraeni Y (2020) Utilization of wood vinegar as plant based insecticide in mulberry (*Morus* sp). In *IOP Conference Series: Materials Science and Engineering*. Vol. 935. 012027. Bristol, UK: IOP Publishing. doi:10.1088/1757-899X/935/1/012027.

[ref11] Douglas AE (1988) Sulphate utilization in an *aphid symbiosis*. *Insect Biochemistry* 18, 599–605. doi:10.1016/0020-1790(88)90012-1.

[ref12] Durand R, Bouvresse S, Andriantsoanirina V, Berdjane Z, Chosidow O and Izri A (2011) High frequency of mutations associated with head lice pyrethroid resistance in schoolchildren from Bobigny, France. *Journal of Medical Entomology* 48(1), 73–75. doi:10.1603/me10115. 21337951

[ref13] El-Fawy MM, Abo-Elyousr KAM, Sallam NMA, El-Sharkawy RMI and Ibrahim YE (2023) Fungicidal effect of guava wood vinegar against *Colletotrichum coccodes* causing black dot disease of potatoes. *Horticulturae* 9, 710. doi:10.3390/horticulturae9060710.

[ref14] Essa RE, Afifi AA, Thalooth AT and El-Ashry SM (2023) Maximizing productivity of some faba bean varieties by foliar of wood vinegar and algae under sandy soil conditions. *Journal of Plant Production* 509–516. doi:10.21608/jpp.2023.242137.1276.

[ref15] Faisal M, Utari S, Hayvia Z and Maulana I (2019) A preliminary study of the utilization of Cu (II) modified liquid smoke to inhibit the activity of white-rot fungi (*Schizophyllum commune Fr*) in a pinewood *in-vitro*. *International Journal of GEOMATE* 17, 56–61. doi:10.21660/2019.61.4679.

[ref16] Flematti GR, Ghisalberti EL, Dixon KW and Trengove RD (2009) Identification of alkyl substituted 2 H-furo [2, 3-c] pyran-2-ones as germination stimulants present in smoke. *Journal of Agricultural and Food Chemistry* 57(20), 9475–9480. doi:10.1021/jf9028128. 19785418

[ref17] Fu YT, Yao C, Deng YP, Elsheikha HM, Shao R, Zhu XQ and Liu GH (2022) Human pediculosis, a global public health problem. *Infectious Diseases of Poverty* 11, 58. doi:10.1186/s40249-022-00986-w. 35619191 PMC9134731

[ref18] Gama GSP, Pimenta AS, Feijó FMC, Santos CSD, Castro RVDO, Azevedo TKBD and De Medeiros LCD (2023) Effect of pH on the antibacterial and antifungal activity of wood vinegar (Pyroligneous extract) from Eucalyptus. *Revista Árvore* 47:e4711. doi:10.1590/1806-908820230000011.

[ref19] Germinara GS, Conte A, Lecce L, Di Palma A and Del Nobile MA (2010) Propionic acid in bio-based packaging to prevent *Sitophilus granarius* (L.) (*Coleoptera, Dryophthoridae*) infestation in cereal products. *Innovative Food Science & Emerging Technologies* 11, 498–502. doi:10.1016/j.ifset.2010.03.001.

[ref20] Germinara GS, Rotundo G and De Cristofaro A (2007) Repellence and fumigant toxicity of propionic acid against adults of *Sitophilus granarius* (L.) and *S. oryzae* (L.). *Journal of Stored Products Research* 43, 229–233. doi:10.1016/j.jspr.2006.06.002.

[ref21] Hashemi SM, Safavi SA and Estaji A (2014) Insecticidal activity of wood vinegar mixed with *Salvia leriifolia* (Benth.) extract against *Lasioderma serricorne* (F.). *Biharean Biologist* 8, 5–11.

[ref22] He Y, Zhang J, Shen L, Wang L, Qian C, Lyu H, Cong Y, Cai J, Chen X, Wen X, Wen C and Cai W (2023) Eugenol derivatives: Strong and long-lasting repellents against both undisturbed and disturbed red imported fire ants. *Journal of Pest Science* 96, 327–344. doi:10.1007/s10340-022-01501-8.

[ref23] Iacomino G, Idbella M, Staropoli A, Nanni B, Bertoli T, Vinale F and Bonanomi G (2024) Exploring the potential of wood vinegar: Chemical composition and biological effects on crops and pests. *Agronomy* 14(1), 114. doi:10.3390/agronomy14010114.

[ref24] Jesu G, Laudonia S, Bonanomi G, Flematti G, Germinara SG, Pistillo M, Giron D, Bèzier A and Vinale F (2022) Biochar-derived smoke waters affect *Bactrocera oleae* behavior and control the olive fruit fly under field conditions. *Agronomy* 12, 2834. doi:10.3390/agronomy12112834.

[ref25] Jesu G, Vinale F, Lorito M and Laudonia S (2024) Trichoderma metabolites 6-pentyl-α-pyrone and harzianic acid affect the reproduction and microbiome of *Bactrocera oleae*. *Journal of Pest Science* doi:10.1007/s10340-024-01796-9.

[ref26] Kiarie-Makara MW, Yoon HS and Lee DK (2010) Repellent efficacy of wood vinegar against *Culex pipiens pallens* and *Aedes togoi* (Diptera: *Culicidae*) under laboratory and semi‐field conditions. *Entomological Research* 40, 97–103. doi:10.1111/j.1748-5967.2010.00265.x.

[ref27] Kim DH, Seo HE, Lee S and Lee K (2008) Effects of wood vinegar mixed with insecticides on the mortalities of *Nilaparvata lugens* and *Laodelphax striatellus* (Homoptera: Delphacidae). *Animal Cells and Systems* 12, 47–52. doi:10.1080/19768354.2008.9647153.

[ref28] Liu X, Zhan Y, Li X, Li Y, Feng X, Bagavathiannan M, Zhang C, Qu M and Yu J (2021) The use of wood vinegar as a non-synthetic herbicide for control of broadleaf weeds. *Industrial Crops and Products* 173, 114105. doi:10.3390/agronomy12123120.

[ref29] Malheiro R, Casal S, Cunha SC, Baptista P and Pereira JA (2016) Identification of leaf volatiles from olive (*Olea europaea*) and their possible role in the ovipositional preferences of olive fly, *Bactrocera oleae* (Rossi) (Diptera: *Tephritidae*). *Phytochemistry* 121, 11–19. doi:10.1016/j.phytochem.2015.10.005. 26603276

[ref30] McNair CM (2015) Ectoparasites of medical and veterinary importance: Drug resistance and the need for alternative control methods. *Journal of Pharmacy and Pharmacology* 67, 351–363. doi:10.1111/jphp.12368. 25644683

[ref31] Mhamdi R (2023) Evaluating the evolution and impact of wood vinegar research: A bibliometric study. *Journal of Analytical and Applied Pyrolysis* 175, 106190. doi:10.1016/j.jaap.2023.106190.

[ref32] Morgoglione ME, Bosco A, Maurelli MP, Alves LC, Saralli G, Bruni G, Cringoli G and Rinaldi L (2020) A 10-year surveillance of *Eimeria* spp. in cattle and buffaloes in a Mediterranean area. *Frontiers in Veterinary Science* 7, 410. doi:10.3389/fvets.2020.00410. 32851006 PMC7417623

[ref33] Neglia G, Veneziano V, De Carlo E, Galiero G, Borriello G, Francillo M, Campanile G, Zicarelli L and Manna L (2013) Detection of *Brucella abortus* DNA and RNA in different stages of development of the sucking louse *Haematopinus tuberculatus*. *BMC Veterinary Research* 9, 1–9. doi:10.1186/1746-6148-9-236. 24289112 PMC4220825

[ref34] Niassy S, Mohamed SA, Cheseto X, Omuse ER, Ochola JB, Khamis F, Badji B, Ndlela S, Ombura FL, Okun NL, Kupesa DM, Dubois T, Belayneh YT, Subramanian S and Ekesi S (2023) Response of some mango-infesting fruit flies to aqueous solutions of the basil plant *Ocimum tenuiflorum* L. *Frontiers in Horticulture* 2, 1139525. doi:10.3389/fhort.2023.1139525.

[ref35] Oramahi HA, Permana RD, Diba F and Indrayani Y (2023) The composition and termicidal activity of vinegar from medang wood (*Cinnamomum* sp.) under different pyrolysis temperature. *Floresta E Ambiente* 30, e20230016. doi:10.1590/2179-8087-FLORAM-2023-0016.

[ref36] Othman N, Elias NH and Zainalabidin N (2023) Wood vinegar as an alternative insecticide in controlling rice weevil, *Sitophylus oryzae* (Coleoptera: *Curculionidae*). *Advances in Agricultural and Food Research Journal* 4(1), doi:10.36877/aafrj.a0000315.

[ref37] Ouattara HAA, Niamké FB, Yao JC (2023)Wood vinegars: Production processes, properties, and valorization. *Forest Products Journal* 73, 239–249. doi:10.13073/FPJ-D-23-00021.

[ref38] Pagnini U, Montagnaro S, Pacelli F, De Martino L, Florio S, Rocco D, Iovane G, Pacilio M, Gabellini C, Marsili S and Giordano A (2004) The involvement of oxidative stress in bovine herpesvirus type 4-mediated apoptosis. *Frontiers in Bioscience* 9, 2106–2114. doi:10.2741/1320. 15353273

[ref39] Palma P, Palma VL, Matos C, Fernandes RM, Bohn A, Soares AMVM and Barbosa IR (2009) Assessment of the pesticides atrazine, endosulfan sulphate and chlorpyrifos for juvenoid-related endocrine activity using *Daphnia magna*. *Chemosphere* 76, 335–340. doi:10.1016/j.chemosphere.2009.03.059. 19403157

[ref40] Paulraj MG, Reegan AD and Ignacimuthu S (2011) Toxicity of benzaldehyde and propionic acid against immature and adult stages of *Aedes aegypti* (Linn.) and *Culex quinquefasciatus*. Say)(Diptera: *Culicidae*. *Journal of Entomology* 8, 539–547. doi:10.3923/je.2011.539.547.

[ref41] Rasheed NOA, Shiha NA, Mohamed SS and Ibrahim WW (2023) SIRT1/PARP-1/NLRP3 cascade as a potential target for niacin neuroprotective effect in lipopolysaccharide-induced depressive-like behavior in mice. *International Immunopharmacology* 123, 110720. doi:10.1016/j.intimp.2023.110720. 37562290

[ref42] Ratajac R, Pavlićević A, Petrović J, Stojanov I, Orčić D and Štrbac F (2024) *In vitro* evaluation of acaricidal efficacy of selected essential oils against *Dermanyssus gallinae*. *Pakistan Veterinary Journal* 44, 93–98. doi:10.29261/pakvetj/2023.123.

[ref43] Santos NC, Silva JE, Santos ACC, Dantas JDO, Tavares SRSA and Andrade VS (2023) Bioactivity of essential oils from Croton grewioides and its major compounds: Toxicity to soybean looper *Chrysodeixis includens* and selectivity to the predatory stink bug *Podisus nigrispinus*. *Environmental Science and Pollution Research* 30, 18798–18809. doi:10.1007/s11356-022-23414-w. 36217049

[ref44] Sarfraz R, Jamil M, Ali M, Ullah M, Jabeen N, Ramzan NJF, Khan A, Khan I, Khan MZ, Shakirullah A, Khan M and Khan I (2023) Identification, prevalence, distribution of ectoparasites and associated host-related risk factors on domestic animals in district Dera. *Pure and Applied Biology (PAB)* 12, 931–938. doi:10.19045/bspab.2023.120094.

[ref45] Scolari F, Valerio F, Benelli G, Papadopoulos NT and Vaníčková L (2021) Tephritid fruit fly semiochemicals: Current knowledge and future perspectives. *Insects* 12, 408. doi:10.3390/insects12050408. 33946603 PMC8147262

[ref46] Shakya M, Kaveri KG, Jamra S, Singh M, Fular A, Agrawal V, Jatav GP, Jayraw AK and Kumar S (2022) Detection of deltamethrin, cypermethrin and flumethrin efficacy against buffalo lice—*Haematopinus tuberculatus*. *Tropical Animal Health and Production* 54, 66. doi:10.1007/s11250-022-03063-4. 35041093

[ref47] Staropoli A, Vassetti A, Salvatore MM, Andolfi A, Prigigallo MI, Bubici G, Scagliola M, Salerno P and Vinale F (2021) Improvement of nutraceutical value of parsley leaves (*Petroselinum crispum*) upon field applications of beneficial microorganisms. *Horticulturae* 7(9), 281. doi:10.3390/horticulturae7090281.

[ref48] Štrbac F, Krnjajić S, Stojanović D, Ratajac R, Simin N, Orčić D, Rinaldi L, Ciccone E, Maurelli MP, Cringoli G and Bosco A (2023) *In vitro* and *in vivo* anthelmintic efficacy of peppermint (*Mentha* x *piperita* L.) essential oil against gastrointestinal nematodes of sheep. *Frontiers in Veterinary Science* 10, 1232570. doi:10.3389/fvets.2023.1232570. 37662995 PMC10472939

[ref49] Veneziano V, Neglia G, Buono F, Pacifico L, Manna L, Bassini A, Miotto L, Santoro M and Gokbulut C (2017) *In vitro* efficacy of alphacypermethrin on the buffalo louse *Haematopinus tuberculatus* (Burmeister, 1839). *Buffalo Bulletin* 36, 353–360.

[ref50] Veneziano V, Neglia G, Cimmino R, Balestrieri A, Rufrano D, Bastianetto E, Santoro M and Gokbulut C (2013) The efficacy and safety of alphacypermethrin as a pour-on treatment for water buffalo (*Bubalus bubalis*) infested with *Haematopinus tuberculatus* (Phthiraptera: *Haematopinidae*). *Parasitology Research* 112, 2907–2912. doi:10.1007/s00436-013-3462-8. 23733232

[ref51] Verderio P, Lecchi M, Ciniselli CM, Shishmani B, Apolone G and Manenti G (2023) 3Rs principle and legislative decrees to achieve high standard of animal research. *Animals* 13, 277. doi:10.3390/ani13020277. 36670818 PMC9854901

[ref52] Yang Z, Bao L, Song W, Zhao X, Liang H, Yu M and Qu M (2024) Nicotinic acid changes rumen fermentation and apparent nutrient digestibility by regulating rumen microbiota in Xiangzhong black cattle. *Animal Bioscience* 37, 240–252. doi:10.5713/ab.23.0149. 37905319 PMC10766483

[ref53] Zicarelli L (2004) Buffalo milk: Its properties, dairy yield and mozzarella production. *Veterinary Research Communications* 28(Suppl 1), 127–135. doi:10.1023/B:VERC.0000045390.81982.4d. 15372941

